# Changes in the expression of DNA-binding/differentiation protein inhibitors in neurons and glial cells of the gerbil hippocampus following transient global cerebral ischemia

**DOI:** 10.3892/mmr.2014.3084

**Published:** 2014-12-11

**Authors:** JAE-CHUL LEE, BAI HUI CHEN, JEONG-HWI CHO, IN HYE KIM, JI HYEON AHN, JOON HA PARK, HYUN-JIN TAE, GEUM-SIL CHO, BING CHUN YAN, DAE WON KIM, IN KOO HWANG, JINSEU PARK, YUN LYUL LEE, SOO YOUNG CHOI, MOO-HO WON

**Affiliations:** 1 Department of Neurobiology, School of Medicine, Kangwon National University, Chuncheon, Gangwon 200-701, Republic of Korea; 2 Department of Physiology, Institute of Neurodegeneration and Neuroregeneration, College of Medicine, Hallym University, Chuncheon, Gangwon 200-702, Republic of Korea; 3 Department of Biomedical Science, Research Institute of Bioscience and Biotechnology, Hallym University, Chuncheon, Gangwon 200-702, Republic of Korea; 4 Department of Neuroscience, College of Medicine, Korea University, Seoul 136-705, Republic of Korea; 5 Institute of Integrative Traditional and Western Medicine, Medical College, Yangzhou University, Yangzhou, Jiangsu 225001, P.R. China; 6 Department of Biochemistry and Molecular Biology, Research Institute of Oral Sciences, College of Dentistry, Kangnung-Wonju National University, Gangneung, Gangwon 210-702, Republic of Korea; 7 Department of Anatomy and Cell Biology, College of Veterinary Medicine, Research Institute for Veterinary Science, Seoul National University, Seoul 151-742, Republic of Korea

**Keywords:** inhibitors of DNA binding proteins, ischemic damage, hippocampus, pyramidal neurons, delayed neuronal death, glial cells

## Abstract

Inhibitors of DNA-binding/differentiation (ID) proteins bind to basic helix-loop-helix (bHLH) transcription factors, including those that regulate differentiation and cell-cycle progression during development, and regulate gene transcription. However, little is known about the role of ID proteins in the brain under transient cerebral ischemic conditions. In the present study, we examined the effects of ischemia-reperfusion (I-R) injury on the immunoreactivity and protein levels of IDs 1–4 in the gerbil hippocampus proper *Cornu Ammonis* regions CA1–3 following 5 min of transient cerebral ischemia. Strong ID1 immunoreactivity was detected in the nuclei of pyramidal neurons in the hippocampal CA1–3 regions; immunoreactivity was significantly changed following I-R in the CA1 region, but not in the CA2/3 region. Five days following I-R, ID1 immunoreactivity was not detected in the CA1 pyramidal neurons. ID1 immunoreactivity was detected only in GABAergic interneurons in the ischemic CA1 region. Weak ID4 immunoreactivity was detected in non-pyramidal cells, and immunoreactivity was again only changed in the ischemic CA1 region. Five days following I-R, strong ID4 immunoreactivity was detected in non-pyramidal cells, which were identified as microglia, and not astrocytes, in the ischemic CA1 region. Furthermore, changes in the protein levels of ID1 and ID4 in the ischemic CA1 region studied by western blot were consistent with patterns of immunoreactivity. In summary, these results indicate that immunoreactivity and protein levels of ID1 and ID4 are distinctively altered following transient cerebral ischemia only in the CA1 region, and that the changes in ID1 and ID4 expression may relate to the ischemia-induced delayed neuronal death.

## Introduction

Transient global cerebral ischemia, due to temporary blood flow deprivation of the brain, causes insidious delayed neuronal degeneration in the hippocampus (1,2). Specifically, the pyramidal neurons in the hippocampal *Cornu Ammonis* region CA1 are the most vulnerable to transient global cerebral ischemia; however, neurons in the CA3 and dentate gyrus remain essentially intact (1,3). Neuronal death in the CA1 region, which occurs several days after ischemia-reperfusion (I-R), is described as ‘delayed neuronal death’ (1). It has been suggested that the molecular events associated with delayed neuronal death are caused by glutamate receptor-mediated neurotoxicity (4), free radical-related damage (5) and oxidative stress (6). However, the precise mechanisms of delayed neuronal death remain unclear.

Inhibitors of DNA binding/differentiation (ID) proteins regulate gene transcription through binding to basic helix-loop-helix (bHLH) transcription factors; four members of this protein family, ID1–4, have been identified in mammals (7–10). Members of the ID protein family share a highly conserved bHLH domain and are similar in size (13–20 kDa), but display extensive sequence variation outside the bHLH domain. As transcription factors, ID proteins are involved in the development of the nervous system, muscle genesis, tumorigenesis, cell cycle regulation and apoptosis (10–12). ID1–3 are expressed in dividing neuroblasts of the central nervous system (CNS) during development (13,14). However, ID4, which is dissimilar to the other ID proteins, is exclusively localized in the regions undergoing neuronal maturation in the CNS and the peripheral nervous system (13,15). Expression of ID proteins is very limited in the adult CNS, but is detectable in distinct populations of adult post-mitotic neurons in specific regions, such as all layers of the cerebral cortex except layer IV, the Purkinje cell layer of the cerebellum, the olfactory bulb (the mitral cell, glomerular and internal granule cell layers), the hippocampus and the suprachiasmatic nucleus in the adult rodent brain (14,16–19).

The roles and the changes in ID protein levels induced by transient cerebral ischemia in the hippocampus have not been studied in detail. Therefore in the present study, we examined the changes in immunoreactivity and protein levels of ID1–4 in the ischemic hippocampus of the gerbil 5 min after transient ischemia; the gerbil has been established as a good model for the study of transient global cerebral ischemia (20–23).

## Materials and methods

### Animals and ethics

Male Mongolian gerbils (*Meriones unguiculatus*) were obtained from the Experimental Animal Center at the Kangwon National University (Chuncheon, Korea). Gerbils were used at 6 months of age (body weight, 65–75 g). The animals were housed in conventional cages at 23°C and 60% humidity, with a 12-h light/12-h dark cycle. The animals had free access to food and water. The procedures for animal handling and care adhered to guidelines that are in compliance with the current international laws and policies (Guide for the Care and Use of Laboratory Animals, The National Academies Press, 8th edition, Washington DC, USA, 2011) and they were approved by the Institutional Animal Care and Use Committee of the Kangwon National University. All experiments were conducted with care to minimize the number and the suffering of animals.

### Induction of transient cerebral ischemia

Cerebral ischemia was established with a method previously described by our group (24,25). Briefly, ischemia was induced by bilateral common carotid artery occlusion under anesthesia by inhalation of 2.5% isoflurane in 30% O_2_ and 70% N_2_. Bilateral common carotid arteries were occluded using non-traumatic aneurysm clips for 5 min. The complete interruption of blood flow was confirmed by observing the central artery in the retinae using an ophthalmoscope. During surgery, the animals were kept on a heating pad at 37±0.5°C. Thereafter, the animals were kept on a thermal incubator (Mirae Medical Foundation Health Improvement Centre, Seoul, Korea) to maintain their body temperature until sacrifice. Sham-operated animals were exposed to similar surgery without carotid artery occlusion.

### Tissue processing for histology

For histological examination, the sham-operated and ischemic gerbils (sham and ischemia groups, n=7 in each) were deeply anesthetized with chloral hydrate (300 mg/kg, intraperitoneal injection) and transcardially perfused with 0.1 M phosphate-buffered saline (PBS; pH 7.4) followed by perfusion with 4% paraformaldehyde in 0.1 M phosphate buffer (pH 7.4) at 12 h, 1, 2, 5 and 10 days after ischemia-reperfusion. Following perfusion, the brains were carefully dissected and post-fixed in 4% paraformaldehyde for 6 h. The brain tissues were cryoprotected by infiltration in 30% sucrose overnight. Next, frozen tissues containing the hippocampus were serially cut into 30-μm-thick slices with a cryostat (CM1900 UV; Leica, Wetzlar, Germany) and placed into six-well plates containing PBS.

### Assessment of neuronal damage

Neuronal damage in the hippocampal CA1 region was examined in the sham and ischemia groups after transient cerebral ischemia by Cresyl violet (CV) staining, neuronal nuclear antigen (NeuN) immunohistochemistry and Fluoro-Jade B (F-J B) histofluorescence at designated time points (1, 2, 5 and 10 days after reperfusion).

### CV staining

To examine neuronal damage in the brain upon transient cerebral ischemia, sections from the sham and the ischemia groups were mounted on gelatin-coated microscopy slides. Cresyl violet acetate (Sigma-Aldrich, St. Louis, MO, USA) was dissolved in distilled water at 1.0% (w/v), and glacial acetic acid was added to this solution. The sections were stained and dehydrated by immersing in serial ethanol baths, and were then mounted with Canada balsam (Kanto Chemical Co., Ltd., Tokyo, Japan).

### NeuN immunohistochemical detection

To investigate the neuronal changes in the CA1 region upon transient cerebral ischemia, the anti-NeuN antibody was used, which targets a neuron-specific soluble nuclear antigen. Briefly, the sections were sequentially treated with 0.3% hydrogen peroxide (H_2_O_2_) in PBS for 30 min and 10% normal goat serum in 0.05 M PBS for 30 min. The sections were next incubated with diluted mouse anti-NeuN (1:1,000; Chemicon International, Temecula, CA, USA) overnight at 4°C. Next, the tissues were exposed to biotinylated goat anti-mouse IgG and streptavidin peroxidase complex (1:200; Vector Laboratories Inc., Burlingame, CA, USA). They were then visualized by addition of 3,3′-diaminobenzidine in a 0.1 M Tris-HCl buffer, and mounted on gelatin-coated slides. Following dehydration, the sections were mounted with Canada balsam (Kanto Chemical Co., Ltd.).

### F-J B histofluorescence staining

F-J B histofluorescence staining procedures were conducted according to the method reported by Candelario-Jalil *et al* (6). Briefly, the sections were first immersed in a solution containing 1% sodium hydroxide in 80% alcohol, followed by immersion in 70% alcohol. They were then transferred to a 0.06% potassium permanganate solution and transferred to a 0.0004% F-J B staining solution (Histo-Chem Inc., Jefferson, AR, USA). After washing, the sections were placed on a slide warmer (~50°C) and then examined using an epifluorescent microscope (Carl Zeiss, Gottingen, Germany) with a blue (450–490 nm) excitation light and a barrier filter. With this method, neurons that undergo degeneration brightly fluoresce in comparison to the background (26).

### Cell counts

In order to ensure objectivity, all measurements were blindly performed by two observers for each experiment, under the same conditions. The studied tissue sections were selected in a 120-μm interval based on anatomical landmarks corresponding to an anteroposterior position −1.4 ~ −1.8 mm from the stereotaxic atlas of the gerbil brain (27), and cell counts were obtained by averaging the counts from 20 sections taken from each animal. NeuN- and F-J B-positive (^+^) cell structures were observed from 3 layers of the hippocampus proper (strata oriens, pyramidal and radiatum) using an AxioM1 light microscope (Carl Zeiss) equipped with a digital camera (Axiocam; Carl Zeiss) connected to a PC monitor. The number of NeuN- and F-J B^+^ cells was counted in a 250×250 μm^2^ area at approximately the center of the CA1 region. Cell counts were obtained by averaging the total cell number from each animal per group.

### Immunohistochemical detection of ID proteins

To obtain accurate immunoreactivity data, sections from the sham-operated and ischemic animals (n=7 at each time point) were used at designated time points (0, 12 h, 1, 2, 5 and 10 days after reperfusion) under the same conditions. Immunohistochemical staining was performed using anti-ID1, -ID2, -ID3 and ID4 primary antibodies (1:200; Santa Cruz Biotechnology, Inc., Santa Cruz, CA, USA). In order to establish the specificity of immunostaining, a negative control test was carried out using pre-blocking with goat serum, instead of the primary antibody. The negative control showed no immunoreactivity in the studied samples.

Twenty sections per animal were selected to quantitatively analyze immunoreactivity for the ID1, ID2, ID3 and ID4. Digital images of the hippocampal region were captured under an AxioM1 light microscope equipped with an Axiocam digital camera connected to a PC monitor. The immunostaining intensities were semi-quantitatively evaluated using the MetaMorph 4.01 digital image analysis software (Universal Imaging Corporation Ltd., Marlow, UK). The level of immunoreactivity was scaled as −, ±, +, or ++, representing no staining ( grey scale value: ≥200), weakly positive (grey scale value: 150–199), moderate (grey scale value: 100–149), or strong (grey scale value: ≤99) staining, respectively, as in (28).

### Double immunofluorescence staining

In order to identify the cell type showing ID1 and ID4 immunoreactivity, the sections were processed at 5 days after surgery inducing ischemia by double immunofluorescence staining. We used rabbit anti-ID1 (1:25; Santa Cruz Biotechnology, Inc.)/goat anti-glutamic acid decarboxylase 67 (GAD67) (1:50; Chemicon International) to detect the γ-aminobutyric-acid (GABA)ergic neurons, rabbit anti-ID4 (1:25; Santa Cruz Biotechnology, Inc.)/mouse anti-glial fibrillary acidic protein (GFAP) (1:200; Chemicon International) to detect the astrocytes, and mouse anti-ionized calcium-binding adapter molecule 1 (Iba-1) (1:200; Wako Pure Chemical Industries, Ltd., Osaka, Japan) in order to detect the microglia. The sections were incubated in the antisera mixture overnight at room temperature. After washing 3 times for 10 min with PBS, the sections were incubated in a mixture of fluorescein isothiocyanate-conjugated anti-rabbit IgG (1:600; Jackson ImmunoResearch, West Grove, PA, USA) and Cy3-conjugated anti-goat or anti-mouse IgG (1:200; Jackson ImmunoResearch) for 2 h at room temperature. The sections were then observed under a confocal microscope (LSM510 META NLO; Carl Zeiss).

### Detection of ID1 and ID4 by western blot analysis

To examine changes in the ID1 and ID4 protein levels in the hippocampal CA1 region upon transient cerebral ischemia, sham-operated and ischemic animals (n=5 at each time point) were used for western blot analysis at designated time points (2 and 5 days after I-R). Following animal sacrifice, the brain was removed and transversely cut into serial sections of 400-μm thickness using a vibratome (Leica); the hippocampal CA1 region was then dissected with a surgical blade. The tissues were homogenized in 50 mmol/l PBS (pH 7.4) containing 0.1 mmol/l ethylene glycol-O-O′-bis(2-amino-ethyl)-N,N,N′,N′-tetraacetic acid (pH 8.0), 0.2% Nonidet P-40, 10 mmol/l ethylenediamime-N,N,N′,N′-tetraacetic acid (pH 8.0), 15 mmol/l sodium pyrophosphate, 100 mmol/l β-glycerophosphate, 50 mmol/l NaF, 150 mmol/l NaCl, 2 mmol/l sodium orthovanadate, 1 mmol/l phenylmethylsulfonyl fluoride and 1 mmol/l dithiothreitol (DTT). Following centrifugation at 22,000 × g, the protein level was determined in the supernatants using a Pierce™ Micro BCA™ Protein Assay kit with bovine serum albumin as the standard (Thermo Fisher Scientific Inc., Waltham, MA, USA).

Aliquots containing 20 μg of total protein were boiled in loading buffer containing 150 mmol/l Tris-HCl (pH 6.8), 3 mmol/l DTT, 6% sodium dodecyl sulphate, 0.3% bromophenol blue and 30% glycerol. The aliquots were then loaded onto a 10% polyacrylamide gel. Following electrophoresis, the gels were transferred onto nitrocellulose transfer membranes (Pall Corp., East Hills, NY, USA). To reduce background staining, the membranes were incubated with 5% non-fat dry milk in PBS containing 0.1% Tween-20 for 45 min, followed by incubation with rabbit anti-ID1 and -ID4 antisera (1:1,000), peroxidase-conjugated goat anti-rabbit IgG (Sigma-Aldrich) and a Pierce™ Enhanced Chemiluminescent (ECL) substrates (32106; Thermo Fisher Scientific Inc.). The western blots were scanned, and densitometric analysis for the quantification of the bands was performed using the Scion Image software (Scion Corp., Frederick, MD, USA), which provided measures of relative optical density (ROD). ROD values were expressed as a percentage; the ROD of the sham group was defined as 100%.

### Statistical analysis

Data were expressed as the mean ± standard error of the mean (SEM). Differences in the mean ROD between groups were statistically evaluated by a one-way analysis of variance (ANOVA) using the SPSS program (IBM, Armonk, NY, USA). P<0.05 was considered to indicate a statistically significant difference.

## Results

### Neuronal cell death

**Figure 1:**
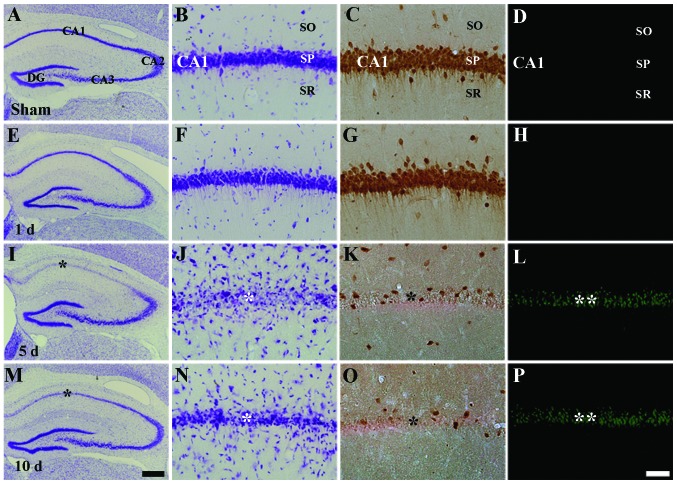
Cresyl violet (CV) staining (first and second columns), neuronal nuclear antigen (NeuN) immunohistochemical staining (third column) and Fluoro-Jade B (F-J B) histofluorescence staining (fourth column) in the *Cornu Ammonis* regions CA1 (A-D) of the sham-operated and (E-P) ischemia groups. In the sham-operated group, numerous CV^+^, NeuN^+^, and no F-J B^+^ cells are detected in the stratum pyramidale (SP). In the ischemia groups, only a few CV^+^ (black asterisks) and NeuN^+^ neurons (arrowhead), and numerous F-J B^+^ cells (white asterisks) are detected in the stratum pyramidale (SP) at 5 and 10 days following ischemia-reperfusion. DG, dentate gyrus; SO, stratum oriens; and SR, stratum radiatum. Scale bar=800 (A, E, I and M) and 50 μm (B-D, F-H, J-L and N-P).

In this study, we examined delayed neuronal death in the hippocampal CA1 region using CV histochemistry, NeuN immunohistochemistry and F-J B histofluorescence staining (Fig. 1). In the sham-operated group, neurons in the stratum pyramidale of the CA1 region were well stained with CV and NeuN, but not with F-J B (Table I, Fig. 1A–D). One and two days following I-R, we did not find any significant change in the number of CV^+^, NeuN^+^ and F-J B^+^ cells in the ischemic CA1 region (Fig. 1E–H). However, 5 days after I-R, CV^+^ cells and NeuN^+^ neurons were significantly decreased in the stratum pyramidale of the CA1 region (Table I, Fig. 1I–K); at this time point, numerous F-J B^+^ cells were observed in the stratum pyramidale of the CA1 region (Table I, Fig. 1L). At 10 days following I-R, the distribution pattern of CV^+^ cells, NeuN^+^ neurons and F-J B^+^ cells in the ischemic CA1 region was similar to that observed at the 5 days post-ischemia (Fig. 1M–P). In the CA2 and CA3 regions, no difference in the distribution pattern of CV^+^ neurons was found between the sham-operated and the ischemia groups (Fig. 1A, E, I and M).

### ID1 immunoreactivity

**Figure 2:**
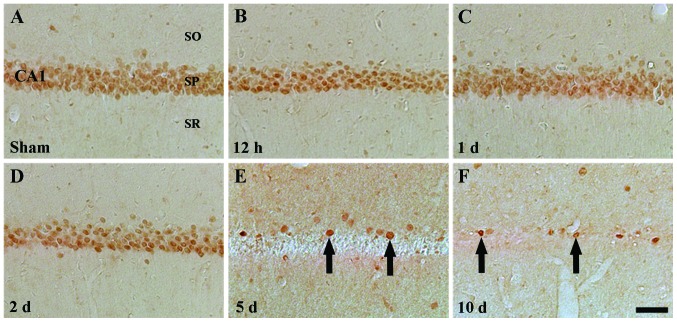
Immunohistochemical detection of ID1 in the *Cornu Ammonis* region CA1 (A) of the sham-operated and (B-F) ischemia groups. ID1 immunoreactivity is easily detected in the stratum pyramidale (SP) in the sham-operated group. ID1 immunoreactivity in the SP is markedly decreased 5 days after ischemia-reperfusion; a few cells near the SP (arrows) show ID1 immunoreactivity. ID, inhibitors of DNA binding/differentiation proteins; SO, stratum oriens; SR, stratum radiatum; h, hours; and d, days. Scale bar=50 μm.

**Figure 3:**
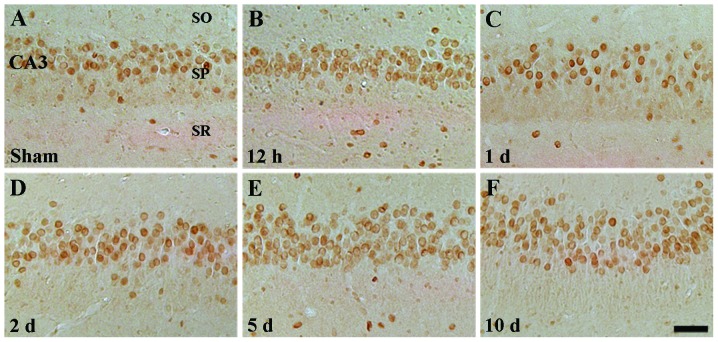
Immunohistochemical detection of ID1 in the *Cornu Ammonis* region CA3 (A) of the sham-operated and (B-F) ischemia groups. In the ischemia groups, ID1 immunoreactivity is not significantly changed compared to that of the sham-operated group. ID, inhibitors of DNA binding/differentiation proteins; SO, stratum oriens; SP, stratum pyramidale; and SR, stratum radiatum; h, hours; and d, days. Scale bar=50 μm.

In the sham-operated group, ID1 immunoreactivity was detected in the pyramidal neurons of the stratum pyramidale in the hippocampal regions CA1–3, especially in the nucleus, but not in the cytoplasm, of the pyramidal neurons (Table II, Figs. 2A and 3A). ID1 immunoreactivity was prominently changed in the pyramidal cells of the CA1 region, and not of the CA2/3 region, following I-R. In the CA1 region of the ischemia groups, positive ID1 immunoreactivity was detected until 2 days post-ischemia (Table II, Fig. 2B–D). Five days following I-R, ID1 immunoreactivity was hardly detectable in the pyramidal neurons of the stratum pyramidale of the CA1 region (Table II, Fig. 2E) and following this time point, the pattern of ID1 immunoreactivity in the CA1 region was similar to that observed 5 days following I-R (Table II, Fig. 2F). At these time points (5 and 10 days), a few ID1^+^ cells were observed near the stratum pyramidale (Fig. 2E and F).

By contrast, ID1 immunoreactivity in the CA2/3 region of the sham-operated group was similar to that observed in the CA1 region (Table II, Fig. 3A). In the ischemia groups, ID1 immunoreactivity was barely changed upon I-R (Table II, Fig. 3B–F).

### ID2 and ID3 immunoreactivity

No ID2^+^ or ID3^+^ cells were detected in the hippocampus proper (CA1-CA3) of both the sham-operated and the ischemia groups (data not shown).

### ID4 immunoreactivity

**Figure 4:**
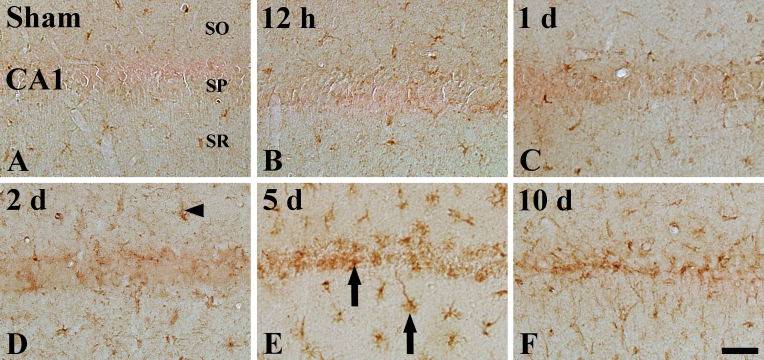
Immunohistochemical detection of ID4 in the *Cornu Ammonis* region CA1 (A) of the sham-operated and (B-F) ischemia groups. ID4 immunoreactivity is weakly detected in the processes (arrow head) of the stratum oriens (SO) and stratum radiatum (SR) in the sham-operated group 2 days after ischemia-reperfusion and is markedly increased from 5 days (arrows) after ischemia-reperfusion. ID, inhibitors of DNA binding/differentiation proteins; SP, stratum pyramidale; h, hours; and d, days. Scale bar=50 μm.

ID4 immunoreactivity in the sham-operated group was weakly detected in the hippocampal CA1 region; ID4 immunoreactivity was detected in cells that had short processes (indicated by arrowhead in Fig. 3A), in the stratum oriens and the stratum radiatum (Table II). In the the CA1 region of the ischemia groups, ID4 immunoreactivity was not changed until 1 day after I-R (Table II, Fig. 4B and C). However, ID4 immunoreactivity was slightly increased 2 days following I-R (Table II, Fig. 4D) and reached its highest level 5 days following I-R (Table II, Fig. 4E). Ten days after I-R, ID4 immunoreactivity was slightly decreased in the ischemic CA1 region, and ID4^+^ processes were longer than those observed at 5 days post-ischemia (Table II, Fig. 4F).

**Figure 5:**
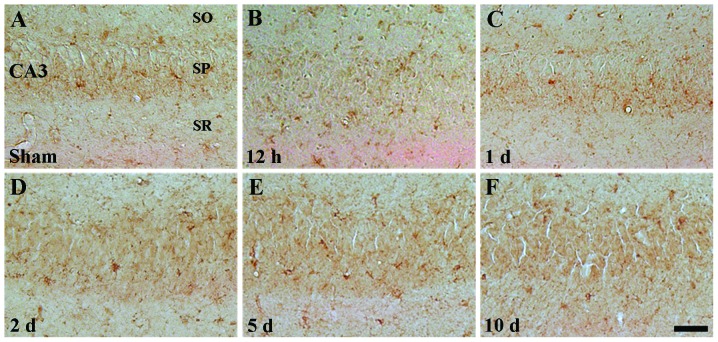
Immunohistochemical detection of ID4 in the *Cornu Ammonis* region CA3 (A) of the sham-operated and (B-F) ischemia groups. In the ischemia groups, ID4 immunoreactivity is not significantly changed compared to the sham-operated group. SO, stratum oriens; SP, stratum pyramidale; SR, stratum radiatum; h, hours; and d, days. Scale bar=50 μm.

Weak ID4 immunoreactivity was also detected in the CA3 region of the sham-operated group (Table II, Fig. 5A). In the ischemia groups, the pattern of ID4 immunoreactivity was similar to that of the sham-operated group (Table II, Fig. 5B–F).

### Colocalization of ID1/GAD67 and ID4/Iba-1

**Figure 6:**
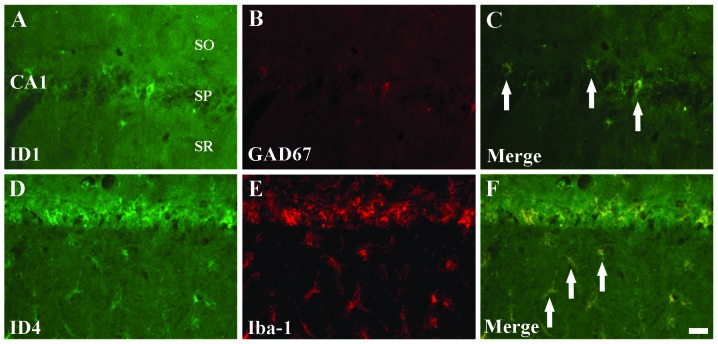
Double immunofluorescence staining for (A) ID1 (green), (B) GAD67 (red), (C) ID1+GAD67 (merged image), (D) ID4 (green), (E) Iba-1 (red) and (F) ID4+Iba-1 (merged image) in the *Cornu Ammonis* region CA1 5 days after ischemia-reperfusion. ID1^+^ cells colocalize with GAD67^+^ GABAergic interneurons (C, arrows); ID4^+^ cells colocalize with GFAP^+^ astrocytes (F, arrows). ID, inhibitors of DNA binding/differentiation proteins; GAD67, glutamic acid decarboxylase 67; GABA, γ-aminobutyric-acid; Iba-1, ionized calcium-binding adapter molecule 1; GFAP, glial fibrillary acidic protein; SO, stratum oriens; SP, stratum pyramidale; and SR, stratum radiatum. Scale bar=20 μm.

ID1 and ID4 immunoreactivities were detected in non-pyramidal cells of the CA1 region, but not in pyramidal neurons, from 5 days post-I-R (Figs. 2E and 4E). In order to identify the cell types of non-pyramidal ID1^+^ and ID4^+^ cells, double immunofluorescence staining was performed for ID1/GAD67, ID4/GFAP and ID4/Iba-1 in the hippocampal CA1 region at 5 days post-ischemia. We found that ID1^+^ cells colocalized with GAD67^+^ GABAergic interneurons (Fig. 6A–C). In addition, ID4^+^ non-pyramidal cells colocalized with Iba-1^+^ microglia, but not with GFAP^+^ astrocytes (data not shown), in the ischemic CA1 region (Fig. 6D–F).

### Changes in ID1 and ID4 protein levels

**Figure 7:**
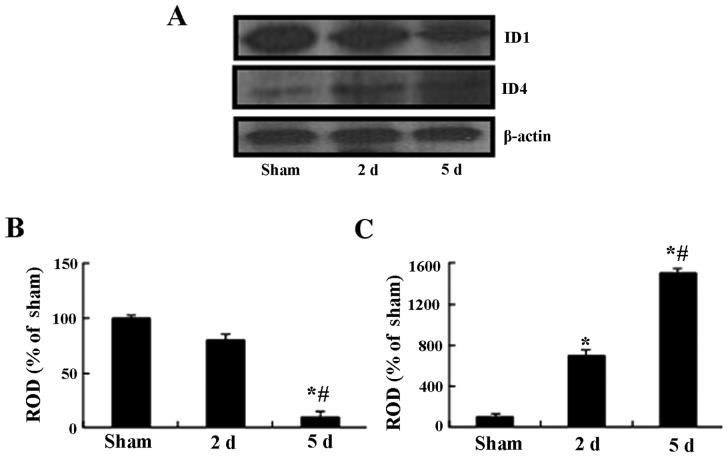
(A) Western blot analysis of ID1 and ID4 in the *Cornu Ammonis* region CA1 of the sham-operated and the ischemia groups, using β-actin as the loading control. The intensity of the immunoblot bands corresponding to (B) ID1 and (C) ID4 was quantified and expressed as relative optical density (ROD)% values relative to the sham-operated group. ^*^P<0.05 compared to the sham-operated group; and ^#^P<0.05 compared to the ischemia group at 2 days (2 d). Bars denote standard error of the mean. ID, inhibitors of DNA binding/differentiation proteins.

Western blot analysis showed that the patterns of changes in the ID1 and ID4 protein levels in the hippocampal CA1 region following I-R were similar to those observed in the immunohistochemical data. In the ischemia groups, the ID1 protein level was slightly decreased at 2 days following I-R compared to that of the sham-operated group; however, the ID1 protein level was significantly decreased 5 days following I-R compared to that of the sham-operated group (Fig. 7). The ID4 protein level was increased compared to the sham-operated group at 2 days following I-R and peaked at 5 days post-I-R (Fig. 7).

## Discussion

In the present study, we observed neuronal damage in the gerbil hippocampal CA1 region 5 days after I-R using NeuN immunohistochemistry and F-J B histofluorescence staining. F-J B has been used as a good fluorescent marker for the detection of neuronal degeneration (26). NeuN^+^ neurons were significantly decreased in the stratum pyramidale of the CA1 region 5 days following I-R, and numerous F-J B^+^ neurons were detected in the stratum pyramidale. This result is consistent with previous studies (1,29).

Mature neurons are terminally-differentiated, post-mitotic cells that do not follow the conventional cell-cycle process. However, a number of studies have shown that in certain neurological disorders, mature neurons reenter the process of cell cycle and undergo cell damage/death including apoptosis (30–32), because they are terminally-differentiated cells that can not re-enter the cell cycle (32,33). There is some evidence that the process of neuronal re-entry into the cell cycle is associated with pathological conditions such as stroke (34,35) and cerebral hypoxia-ischemia (36). ID proteins can inhibit the expression of cyclin-dependent inhibitors such as p21 via inhibition of bHLH factors (10,37). Moreover, the inhibition of ID protein synthesis by antisense oligonucleotides prevents the re-entry of G0-arrested fibroblasts into the cell cycle (38,39). These reports strongly suggest that ID proteins play a role in the G0-S phase transition of the cell cycle. To the best of our knowledge, no studies of the changes in immunoreactivity of ID proteins following ischemic damage have been reported yet, and the roles of ID in the ischemic brain remain unclear. The present study demosntrated a method by which to maintain neurons in the quiescent G0 phase and to protect neurons from damage caused by cerebral ischemia. In this study, we observed changes in ID1 immunoreactivity in the CA1 region following I-R; ID1 immunoreactivity in the pyramidal neurons was markedly decreased from 5 days after I-R, and the changes in the ID1 protein level were in accordance with ID1 immunoreactivity. This finding indicates that ID1 protein may be related to the delay of damage/death of the CA1 pyramidal neurons following I-R injury. It is notable that ID1 immunoreactivity was not distinctively changed in the CA3 region following I-R. The CA3 region is known as the most tolerant area of the hippocampus to ischemia (1,29). In addition, we observed that ID1^+^ cells that survived near the stratum pyramidale 5 days following I-R were GABAergic interneurons. This is the first study to show that ID1 is expressed in the GABAergic interneurons of the CA1 region following transient cerebral ischemia. It has been previously reported that most of GABAergic interneurons in the CA1 region are resistant to transient cerebral ischemia (40,41). Our findings indicate that the ID1 protein may be involved in the survival of GABAergic neurons following an ischemic insult.

In the present study, we found that ID4 immunoreactivity is gradually increased in the microglia in the CA1 region, and peaks 5 days following I-R. This is also the first study reporting expression of ID4 in the microglia, but not astrocytes, in the CA1 region. It is well known that ID4 plays an important role in proliferation and differentiation in a variety of cell types, including neurons (42–44). In addition, it was reported that ID4 protein enhances the translation of mRNAs encoding pro-angiogenic cytokines, such as interleukin 8 and growth-regulated oncogene-α, and that ID4 increases the angiogenic potential of cancer cells (45). Based on these studies, we suggest that ID4 plays a role in proliferation of the microglia following ischemic damage. We do not have a solid hypothesis to explain the increased expression of ID4 in the microglia following I-R, but it is well known that microglia are hyperactivated and hypertrophied in all the layers of the CA1 region following transient cerebral ischemia and before the occurence of delayed neuronal death in the CA1 pyramidal neurons (46). Furthermore, numerous studies have shown that the activation of microglia is associated with the development of neuronal death during ischemia (47–49); microglia secrete large amounts of cytotoxic and inflammatory mediators (50–52), and contribute to the elimination of deleterious debris, promotion of tissue repair and return to tissue homeostasis (53–55). Therefore, it can be postulated that ID4 plays an important role in microglial proliferation in response to ischemic damage. Additional studies are needed to elucidate the exact role of ID4 in the microglia following transient cerebral ischemia.

In summary, immunoreactivity and the protein level of ID1 protein were apparently altered in the CA1 pyramidal neurons following transient cerebral ischemia, while ID4 immunoreactivity was detected in the microglia, but not the astrocytes, in the CA1 region following I-R. These results indicate that changes in the expression of ID1 and ID4 may be associated with delayed neuronal death and glial activation in the CA1 region following ischemic damage.

## Figures and Tables

**Table I:** Changes in the mean number of positively-stained cells in the pyramidal neurons of the ischemic hippocampal *Cornu Ammonis* region CA1 of the gerbil.

	Positive cells	
	
Time after I-R	NeuN^+^	F-J B^+^
Sham	364±17.74	0
5 d	39±10.36^a^	207±28.66^a^

The mean ± standard error of the mean values of neuronal nuclear antigen (NeuN)^+^ and Fluoro-Jade B (F-J B)^+^ cells, counted in a 250×250 μm^2^ area of the stratum pyramidale in the CA1 region after ischemia-reperfusion (I-R). N=7/group.

a P<0.05, significantly different from the sham-operated (sham) group; and d, days.

**Table II:** Semi-quantitative analysis of ID1 and ID4 immunoreactivity in cells of the *Cornu Ammonis* regions CA1 and CA3 of the sham-operated and ischemia groups.

			Time after ischemia/reperfusion					
			
Antibody	Region	Cell type	Sham	12 h	1 d	2 d	5 d	10 d
ID1	CA1	CSP	++	++	++	+	−	±
ID1	CA1	CSOR	±	±	±	±	++	++
ID1	CA3	CSP	++	++	++	++	++	++
ID1	CA3	CSOR	++	++	++	++	++	++
ID4	CA1	CSP	±	±	±	±	±	±
ID4	CA1	CSOR	+	+	+	++	++	++
ID4	CA3	CSP	±	±	±	±	±	±
ID4	CA3	CSOR	+	+	+	+	+	+

Immunoreactivity is scaled as −, ±, + or ++ representing no staining, weakly positive, moderate and strong staining, respectively. ID, inhibitors of DNA binding/differentiation proteins; h, hours; d, days; CSP, cells in stratum pyramidale; and CSOR, cells in stratum oriens and radiatum.

## References

[1] Kirino T (1982). Delayed neuronal death in the gerbil hippocampus following ischemia. Brain Res.

[2] Lin CS, Polsky K, Nadler JV, Crain BJ (1990). Selective neocortical and thalamic cell death in the gerbil after transient ischemia. Neuroscience.

[3] Pulsinelli WA, Brierley JB, Plum F (1982). Temporal profile of neuronal damage in a model of transient forebrain ischemia. Ann Neurol.

[4] Won MH, Kang T, Park S (2001). The alterations of N-methyl-D-aspartate receptor expressions and oxidative DNA damage in the CA1 area at the early time after ischemia-reperfusion insult. Neurosci Lett.

[5] Rastogi L, Godbole MM, Ray M (2006). Reduction in oxidative stress and cell death explains hypothyroidism induced neuroprotection subsequent to ischemia/reperfusion insult. Exp Neurol.

[6] Candelario-Jalil E, Alvarez D, Merino N, Leon OS (2003). Delayed treatment with nimesulide reduces measures of oxidative stress following global ischemic brain injury in gerbils. Neurosci Res.

[7] Kee Y, Bronner-Fraser M (2005). To proliferate or to die: role of Id3 in cell cycle progression and survival of neural crest progenitors. Genes Dev.

[8] Massari ME, Murre C (2000). Helix-loop-helix proteins: regulators of transcription in eucaryotic organisms. Mol Cell Biol.

[9] Perk J, Iavarone A, Benezra R (2005). Id family of helix-loop-helix proteins in cancer. Nat Rev Cancer.

[10] Ruzinova MB, Benezra R (2003). Id proteins in development, cell cycle and cancer. Trends Cell Biol.

[11] Kremer D, Aktas O, Hartung HP, Kury P (2011). The complex world of oligodendroglial differentiation inhibitors. Ann Neurol.

[12] Norton JD (2000). ID helix-loop-helix proteins in cell growth, differentiation and tumorigenesis. J Cell Sci.

[13] Jen Y, Manova K, Benezra R (1997). Each member of the Id gene family exhibits a unique expression pattern in mouse gastrulation and neurogenesis. Dev Dyn.

[14] Neuman T, Keen A, Zuber MX, Kristjansson GI, Gruss P, Nornes HO (1993). Neuronal expression of regulatory helix-loop-helix factor Id2 gene in mouse. Dev Biol.

[15] Riechmann V, Sablitzky F (1995). Mutually exclusive expression of two dominant-negative helix-loop-helix (dnHLH) genes, Id4 and Id3, in the developing brain of the mouse suggests distinct regulatory roles of these dnHLH proteins during cellular proliferation and differentiation of the nervous system. Cell Growth Differ.

[16] Andres-Barquin PJ, Hernandez MC, Israel MA (2000). Id genes in nervous system development. Histol Histopathol.

[17] Elliott RC, Khademi S, Pleasure SJ, Parent JM, Lowenstein DH (2001). Differential regulation of basic helix-loop-helix mRNAs in the dentate gyrus following status epilepticus. Neuroscience.

[18] Rubenstein JL, Anderson S, Shi L, Miyashita-Lin E, Bulfone A, Hevner R (1999). Genetic control of cortical regionalization and connectivity. Cereb Cortex.

[19] Tzeng SF, de Vellis J (1998). Id1, Id2 and Id3 gene expression in neural cells during development. Glia.

[20] Fukuchi T, Katayama Y, Kamiya T, McKee A, Kashiwagi F, Terashi A (1998). The effect of duration of cerebral ischemia on brain pyruvate dehydrogenase activity, energy metabolites and blood flow during reperfusion in gerbil brain. Brain Res.

[21] Lorrio S, Negredo P, Roda JM, Garcia AG, Lopez MG (2009). Effects of memantine and galantamine given separately or in association, on memory and hippocampal neuronal loss after transient global cerebral ischemia in gerbils. Brain Res.

[22] Zhang YB, Kan MY, Yang ZH (2009). Neuroprotective effects of N-stearoyltyrosine on transient global cerebral ischemia in gerbils. Brain Res.

[23] Wang Q, Sun AY, Pardeike J, Muller RH, Simonyi A, Sun GY (2009). Neuroprotective effects of a nanocrystal formulation of sPLA_2_ inhibitor PX-18 in cerebral ischemia/reperfusion in gerbils. Brain Res.

[24] Hwang IK, Eum WS, Yoo KY (2005). Copper chaperone for Cu,Zn-SOD supplement potentiates the Cu,Zn-SOD function of neuroprotective effects against ischemic neuronal damage in the gerbil hippocampus. Free Radic Biol Med.

[25] Park OK, Yoo KY, Lee CH (2010). Arylalkylamine N-acetyltransferase (AANAT) is expressed in astrocytes and melatonin treatment maintains AANAT in the gerbil hippocampus induced by transient cerebral ischemia. J Neurol Sci.

[26] Schmued LC, Hopkins KJ (2000). Fluoro-Jade B: A high affinity fluorescent marker for the localization of neuronal degeneration. Brain Res.

[27] Loskota WJ, Lomax LP, Verity MA, Loskota William James, Lomax Peter, Verity M Anthony (1974). A stereotaxic atlas of the Mongolian gerbil brain (Meriones unguiculatus).

[28] Lee CH, Park JH, Choi JH, Yoo KY, Ryu PD, Won MH (2011). Heat shock protein 90 and its cochaperone, p23, are markedly increased in the aged gerbil hippocampus. Exp Gerontol.

[29] Lee CH, Moon SM, Yoo KY (2010). Long-term changes in neuronal degeneration and microglial activation in the hippocampal CA1 region after experimental transient cerebral ischemic damage. Brain Res.

[30] Nguyen MD, Boudreau M, Kriz J, Couillard-Despres S, Kaplan DR, Julien JP (2003). Cell cycle regulators in the neuronal death pathway of amyotrophic lateral sclerosis caused by mutant superoxide dismutase 1. J Neurosci.

[31] Vincent I, Rosado M, Davies P (1996). Mitotic mechanisms in Alzheimer’s disease?. J Cell Biol.

[32] Byrnes KR, Faden AI (2007). Role of cell cycle proteins in CNS injury. Neurochem Res.

[33] Broughton BR, Reutens DC, Sobey CG (2009). Apoptotic mechanisms after cerebral ischemia. Stroke.

[34] Rashidian J, Iyirhiaro GO, Park DS (2007). Cell cycle machinery and stroke. Biochim Biophys Acta.

[35] Wen Y, Yang S, Liu R, Brun-Zinkernagel AM, Koulen P, Simpkins JW (2004). Transient cerebral ischemia induces aberrant neuronal cell cycle re-entry and Alzheimer’s disease-like tauopathy in female rats. J Biol Chem.

[36] Wang F, Corbett D, Osuga H (2002). Inhibition of cyclin-dependent kinases improves CA1 neuronal survival and behavioral performance after global ischemia in the rat. J Cereb Blood Flow Metab.

[37] Yokota Y, Mori S (2002). Role of Id family proteins in growth control. J Cell Physiol.

[38] Hara E, Yamaguchi T, Nojima H (1994). Id-related genes encoding helix-loop-helix proteins are required for G1 progression and are repressed in senescent human fibroblasts. J Biol Chem.

[39] Barone MV, Pepperkok R, Peverali FA, Philipson L (1994). Id proteins control growth induction in mammalian cells. Proc Natl Acad Sci USA.

[40] Fukuda T, Nakano S, Yoshiya I, Hashimoto PH (1993). Persistent degenerative state of non-pyramidal neurons in the CA1 region of the gerbil hippocampus following transient forebrain ischemia. Neuroscience.

[41] Tortosa A, Ferrer I (1993). Parvalbumin immunoreactivity in the hippocampus of the gerbil after transient forebrain ischaemia: a qualitative and quantitative sequential study. Neuroscience.

[42] Benezra R, Davis RL, Lockshon D, Turner DL, Weintraub H (1990). The protein Id: a negative regulator of helix-loop-helix DNA binding proteins. Cell.

[43] Nagata Y, Todokoro K (1994). Activation of helix-loop-helix proteins Id1, Id2 and Id3 during neural differentiation. Biochem Biophys Res Commun.

[44] Lyden D, Young AZ, Zagzag D (1999). Id1 and Id3 are required for neurogenesis, angiogenesis and vascularization of tumour xenografts. Nature.

[45] Fontemaggi G, Dell’Orso S, Trisciuoglio D (2009). The execution of the transcriptional axis mutant p53, E2F1 and ID4 promotes tumor neo-angiogenesis. Nat Struct Mol Biol.

[46] Sugawara T, Lewen A, Noshita N, Gasche Y, Chan PH (2002). Effects of global ischemia duration on neuronal, astroglial, oligodendroglial and microglial reactions in the vulnerable hippocampal CA1 subregion in rats. J Neurotrauma.

[47] Hailer NP, Jarhult JD, Nitsch R (1996). Resting microglial cells in vitro: analysis of morphology and adhesion molecule expression in organotypic hippocampal slice cultures. Glia.

[48] Hwang IK, Yoo KY, Kim DW (2006). Ionized calcium-binding adapter molecule 1 immunoreactive cells change in the gerbil hippocampal CA1 region after ischemia/reperfusion. Neurochem Res.

[49] Schwartz M, Butovsky O, Bruck W, Hanisch UK (2006). Microglial phenotype: is the commitment reversible?. Trends Neurosci.

[50] Colton CA, Gilbert DL (1987). Production of superoxide anions by a CNS macrophage, the microglia. FEBS Lett.

[51] Han HS, Qiao Y, Karabiyikoglu M, Giffard RG, Yenari MA (2002). Influence of mild hypothermia on inducible nitric oxide synthase expression and reactive nitrogen production in experimental stroke and inflammation. J Neurosci.

[52] Suzuki S, Tanaka K, Nogawa S (1999). Temporal profile and cellular localization of interleukin-6 protein after focal cerebral ischemia in rats. J Cereb Blood Flow Metab.

[53] Hashimoto M, Nitta A, Fukumitsu H, Nomoto H, Shen L, Furukawa S (2005). Involvement of glial cell line-derived neurotrophic factor in activation processes of rodent macrophages. J Neurosci Res.

[54] Laurenzi MA, Arcuri C, Rossi R, Marconi P, Bocchini V (2001). Effects of microenvironment on morphology and function of the microglial cell line BV-2. Neurochem Res.

[55] Lu YZ, Lin CH, Cheng FC, Hsueh CM (2005). Molecular mechanisms responsible for microglia-derived protection of Sprague-Dawley rat brain cells during in vitro ischemia. Neurosci Lett.

